# Early marathon running metrics from inertial measurement units predict significant pace reduction

**DOI:** 10.3389/fspor.2025.1681444

**Published:** 2025-10-17

**Authors:** Yosuke Miyazaki, Hidetoshi Matsui, Kodayu Zushi, Takumi Fukui

**Affiliations:** ^1^ Institute of Sport Science, ASICS Corporation, Kobe, Japan; ^2^ Faculty of Data Science, Shiga University, Hikone, Japan; ^3^ Faculty of Education, Wakayama University, Wakayama, Japan; ^4^ Data Science and AI Innovation Research Promotion Center, Shiga University, Hikone, Japan

**Keywords:** hitting the wall, inertial measurement unit, marathon, pacing strategy, functional data analysis

## Abstract

Marathon runners occasionally experience significant pace reduction in the latter stages of races, a phenomenon known as “hitting the wall”. This study aimed to develop an interpretable model to predict this performance decline using biomechanical variables collected during the early stages of marathons. We analyzed data from 1,437 runners collected during official marathon events held in Japan from August 2022 to May 2025. Biomechanical variables were measured using inertial measurement unit attached to the runners’ lower back. “Hitting the wall” was defined as maintaining a pace exceeding 125% of the average pace from 5 to 20 km continuously for more than 5 km after the 25 km point. Conversely, runners were classified as “NOT hitting the wall” if their pace remained less than 110% of the average pace for more than 10 km. Cases not meeting either criterion were excluded from analysis, resulting in 306 positive cases and 359 negative cases. We applied functional principal component analysis to efficiently handle time-series data and developed a functional logistic regression model using data from the first half of marathons to predict the severe pace reduction. Our model achieved 73.9% accuracy, 75.8% recall, and 70.1% precision. Analysis of coefficient functions in the functional logistic regression model revealed that step length, ground contact time, and vertical stiffness were the strongest predictors of subsequent performance decline. The identified biomechanical signatures could inform personalized training strategies aimed at preventing the “hitting the wall” phenomenon during marathon races.

## 1 Introduction

Recent advancements in wearable sensing technologies have enabled the acquisition of valuable data for performance evaluation and conditioning monitoring across various sports. In marathon running, for example, many participants now routinely employ running-watches and mobile applications to track pace and vital metrics including heart rate during both training and competition. These data allow runners to objectively assess their performance metrics over time. While several approaches to running data utilization have been proposed and investigated (1, 2), there is still room for practical use of data so that runners can achieve their goals more effectively.

To optimize marathon performance, pacing strategy is one of the most critical factors. Even pace and negative split, where speed increases in the latter half of the race, are commonly recommended to maximize performance (3). Even experienced runners who fail to maintain consistent pacing tend to record finishing times that fall short of their personal bests (4). Furthermore, approximately two in five recreational runners experience severe pace reduction and it results in more than 30 min of performance loss compared to their personal records (5, 6). This significant slowing down in pace, which is commonly known as “hitting the wall”, typically occurs around 30 km/20 mile from the start line. Marathon runners thus face a fundamental dilemma between the ambition of achieving personal goals and the risk of “hitting the wall”. To overcome this dilemma, runners need reliable methods to predict potential performance decline based on their physical condition early in the race.

Some studies have investigated changes during or pre- and post-marathon races or prolonged running through blood markers (7, 8) and respiratory gases (9–11). The main physiological cause of the severe pace reduction is thought to be glycogen depletion (12–14). In particular, the glycogen depletion occurs faster at higher intensity during submaximal exercise (15). It is well known that the relative contribution of energy substrates is dependent on exercise intensity, and thus some metrics of exercise intensity during the first half of races likely affect performance deterioration in the latter half. However, measuring blood markers and respiratory gases during races is not easy for most runners. Wearable devices offer a more accessible alternative, providing real-world data without interfering with natural running conditions. In running analytics, two common wearable technologies have emerged: running-watches that primarily collect cardiovascular metrics (e.g., heart rate, heart rate variability), and Inertial Measurement Units (IMUs) that can measure biomechanical variables. Although many of the physiological metrics cannot be directly measured by either running-watches or IMUs, IMU-derived metrics can correlate with oxygen uptake, as demonstrated by Linkis et al. (16). During prolonged running, neuromuscular fatigue and altered motor patterns emerge (17, 18). As fatigue accumulates, runners often modify their running mechanics to compensate for reduced force production capability, leading to changes in stride characteristics and joint kinematics (19). These compensatory movement patterns, while initially allowing maintained performance, may lead to decreased running economy and eventually contribute to more rapid onset of exhaustion (20). Such fatigue-induced alterations in running patterns have been quantitatively assessed using wearable sensor data, with one study reporting that composite indices reflecting atypical running patterns for variables like cadence, pelvic drop, and ground contact time became significantly higher starting at 20–22 km during a marathon (21). Furthermore, Prigent et al. (22) found that biomechanical variables showed significant alterations earlier than heart rate dynamics, potentially providing earlier warning signs of “hitting the wall”. Additionally, biomechanical variables offer the advantage of translating directly into actionable feedback that runners can implement through daily trial and error. Although several researches have attempted to predict fatigue levels or performance decline using data derived from wearable devices (23–27), existing models suffer from limited sample sizes, subject-specific parameters, or focus on highly controlled conditions that may not generalize to real-world marathon settings. While subject-specific parameters can potentially increase prediction accuracy for experienced runners with sufficient data, many recreational runners may not have adequate data representing both of fresh and fatigued states in real-world race conditions. Moreover, few studies have specifically addressed the prediction of “hitting the wall” phenomenon using interpretable models that provide meaningful insights into the underlying biomechanical mechanisms.

Time series data from wearable devices presents unique analytical challenges, particularly when attempting to capture temporal characteristics and patterns. Functional Data Analysis (FDA) offers a powerful framework for analyzing such continuous, time-varying data (28). Traditional biomechanical analyses often focus on discrete features such as peak values or measurements at specific time points, potentially missing important information contained in the complete time series data. In contrast, FDA preserves temporal patterns within the data while reducing dimensionality and mitigating noise. A systematic review by Dannenmaier et al. (29) highlighted FDA's potential to provide detailed and comprehensive insights into biomechanical data. Recent applications of FDA in biomechanical research demonstrate its effectiveness in capturing complex temporal patterns. For example, Doi et al. (30) applied FDA to running form data from IMUs to capture fatigue-related changes as coherent information encompassing the entire running cycle. Similarly, Son et al. (31) utilized FDA to comprehensively analyze jump/landing movements as continuous curves across the complete stance phase.

The primary aim of this study was to develop an interpretable model to predict significant pace reduction in the latter half of a full marathon based on biomechanical variables observed during the first half of the race. By applying FDA techniques to IMU-derived data collected under real marathon conditions, we sought to identify early biomechanical signatures that precede “hitting the wall”. This approach not only addresses the practical need for predictive tools accessible to recreational runners but also contributes to the theoretical understanding of how biomechanical alterations evolve throughout a marathon and their relationship to performance outcomes.

## 2 Methods

The dataset of running form consisted of a total of 1,437 runners (1,277 males and 160 females) measured in full marathon races held in Japan from August 2022 to May 2025. The mean age of participants was 50.4 ± 8.6 years. The average finishing time recorded was 256 ± 44 min. The data were collected as part of the regular service provided by Runmetrix®, where runners voluntarily used a commercially available IMU (CMT-S20R-AS; CASIO COMPUTER Co., LTD.) device during their marathon races. All participants provided consent for their data to be used for research purposes through the service agreement.

The IMU sensor was securely clipped to the back of the runner's shorts. This IMU features a body-worn 9-axis sensor including an accelerometer, gyroscope, magnetometer, and GPS. The sampling frequency was set at 200 Hz, and the measured data were transmitted by the Runmetrix® mobile application. The application contains built-in algorithms that process the raw IMU signals to calculate various running form variables. These running form variables include pace [s/km], cadence [steps/m], step length [% height], pelvic backward lean [deg], vertical motion [% height], body drop [% height], pelvic drop [deg], pelvic elevation [deg], pelvic rotation[deg], pelvic rotation timing, horizontal impact force[m/s^2^], kicking phase duration [ms], ground contact time[% gait cycle], landing impact [m/s^2^], kicking acceleration, [m/s^2^] amount of braking [m/s], and vertical stiffness [kN/m/kg] (32). These variables were calculated for every step and averaged over distances of 250 m, 500 m or 1,000 m, depending on user's settings.

**Figure 1:**
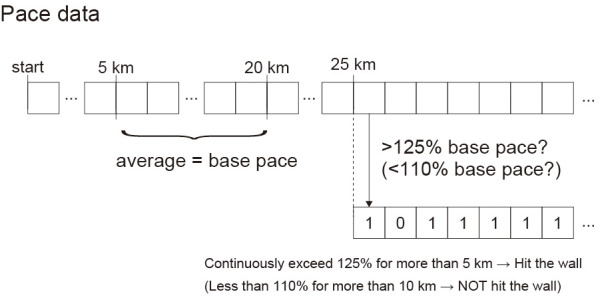
Rule of the classification of the pace label.

For this dataset, we developed an operational definition for identifying “hitting the wall” (HTW) based on the pace profiles (Figure 1), drawing upon methodologies from previous studies (5, 6). This phenomenon was assessed by analyzing the degree of slowdown during the latter stages of the race. Specifically, we defined the average pace from 5 to 20 km as the “base pace”, excluding the initial 5 km segment as pacing can be erratic during the very early stages of a marathon. The “relative pace” for segments after 25 km was calculated as the ratio of the segment pace to the base pace. A race was then classified as “hit the wall” if the relative pace profile continuously exceeded 1.25 (indicating a slowdown of more than 25% compared to the base pace) for more than 5 km. This criterion aligns with the operational definition adopted by Smyth (6), which was established through a sensitivity analysis on a large-scale dataset of over 4 million race records. Smyth (6) also notes that these thresholds are comparable to similar criteria proposed by Berndsen et al. (5), which included slowdowns of approximately 17% over more than 5 km. On the other hand, a race was labeled as “NOT hit the wall” if the relative pace was consistently less than 1.10 for more than 10 km, indicating that the runner largely maintained their pace throughout the race. This threshold is grounded in the general understanding in pacing studies that any pace variation within 10% of the mean race pace is considered to be maintaining pace. A continuous duration of more than 10 km with such a minimal slowdown suggests a sustained and controlled effort. Consequently, races that do not fit either criterion were excluded from the data. For these labels, we considered constructing the prediction model that classifies whether the runner hits the wall.

**Figure 2:**
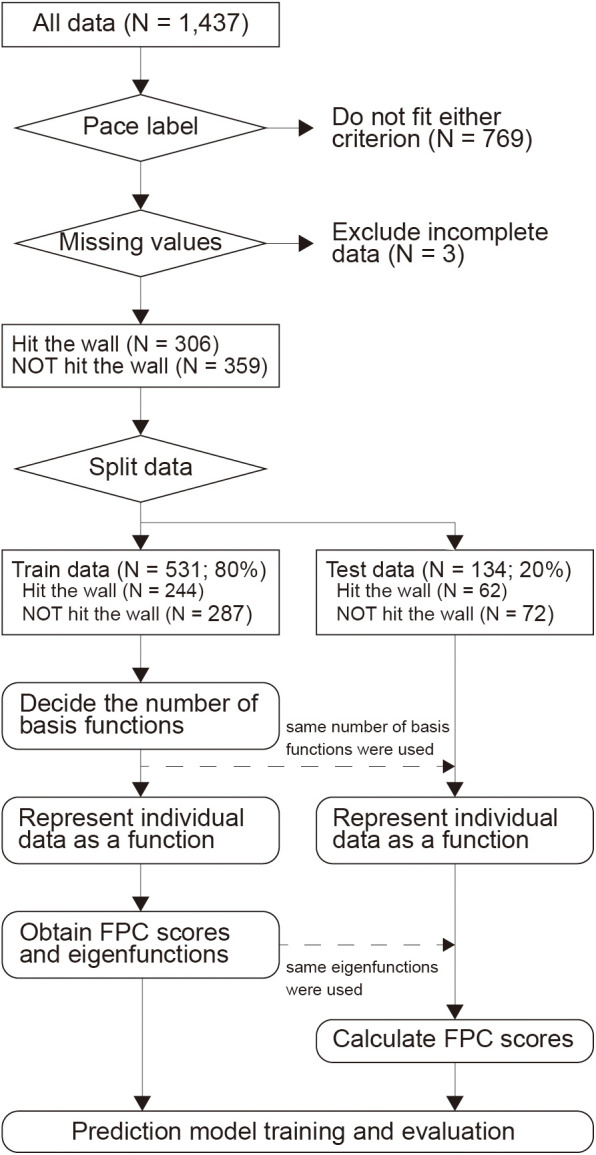
Flowchart of the data analysis.

After the pace labels were classified, data preprocessing was conducted as shown in Figure 2. Data with missing values in any running form variables was excluded from further analysis. The whole dataset was split into training (80%) and test (20%) sets using stratified random sampling to maintain the same distribution of gender, finish time, age, and pace labels in both sets. Before applying the classification model, we utilized functional principal component analysis (FPCA) on the time series data, measured in the races. FPCA can represent the time series data in a low dimensional form. To apply FPCA, the measured time series data were smoothed by using B-spline basis functions (28). The number of basis functions was determined using generalized cross-validation for each variable, and the data for each individual were subsequently represented as a function. This functional representation allowed us to handle data with different sampling intervals in a unified manner. Before applying FPCA, the functional data were standardized by subtracting the mean and dividing by the standard deviation calculated from the training dataset. The same standardization parameters from the training data were applied to the test dataset to ensure consistent scaling across both sets. Then, FPCA was applied to the functional data and we can obtain functional principal component (FPC) scores that represent the feature of the function by a low-dimensional vector. The number of FPCs was determined based on the 99% variance explained in the training dataset. Using FPCA, $x_{ij}(t)$,which is functional data for the *j*-th running form variable of *i*-th runner at distance t in the domain *T*, can be expressed by

$$\begin{array}{c} x_{ij}(t)\;=\;{\hat{\mu }}_{j}(t)\;+\;\overset{K}{\underset{k=1}{\sum }}\;z_{ijk}u_{jk}(t)=\;{\hat{\mu }}_{j}(t)\;+\;z_{ij}^{T}u_{j}(t)\; \end{array}$$

where ${\hat{\mu }}_{j}(t)$ is a sample average function of functional data $x_{ij}(t)\;(i=1,\cdots ,n)$, *K* is the number of FPC, $z_{ij}\;\;=\;(z_{ij1},\ldots ,z_{ijK}{)}^{T}$ is a vector of FPC scores, $u_{j}(t)\;\;=\;(u_{\;j1}(t),\ldots ,u_{jK}(t){)}^{T}$ is a vector of eigenfunctions, and superscript *T* denotes the transpose. The FPCA scores of test data were calculated based on the mean functions and eigenfunctions derived from the FPCA results of the training data.

Prior to constructing the prediction model, we examined multicollinearity among the FPC scores in the training dataset using variance inflation factors (VIF) and correlation coefficients. Variables with VIF > 10 and absolute correlation coefficients > 0.7 were considered for removal to maintain model interpretability while avoiding severe multicollinearity. Based on these criteria, we removed pace, body drop, pelvic drop, and kicking phase duration from the predictor variables. After removing these variables, we confirmed that all remaining variables showed VIF < 7, ensuring both model interpretability and statistical validity. Using the time series datasets transformed into functional data as predictors and the class labels whether the runner hits the wall as a response, we constructed the following functional logistic regression model.

$$\begin{array}{c} \log\frac{\pi_{i}}{1-\pi_{i}}\;=\;\beta_{0}\;+\;\overset{J}{\underset{\;j=1}{\sum }}\;\underset{T}{\int }\;x_{ij}(t)\beta_{j}(t)dt\; \end{array}$$

where $\pi_{i}$ is a probability that the runner hits the wall for the *i*-th runner given the functional data for the running form variables, $\beta_{0}$ is an intercept, $\beta_{j}(t)$ is coefficient function for the *j*-th variable, and *J* is the number of variables. The coefficient function $\beta_{j}(t)$ represents how the functional data $x_{ij}(t)\;(i=1,\ldots ,n)$ relate to the classification at arbitrary distance point *t*. In addition, we suppose that the coefficient function $\beta_{j}(t)$ is represented by basis expansions as

$$\begin{array}{c} \beta_{j}(t)=\overset{K}{\underset{k=1}{\sum }}\;b_{jk}u_{jk}(t)=b_{j}^{T}u_{j}(t) \end{array}$$

where $b_{j}=(b_{\;j1},\ldots ,b_{jK}{)}^{T}$ is a vector of unknown parameters. Then, using the fact that the eigenfunctions $u_{\;j1}(t),\ldots ,u_{jK}(t)$ are orthonormal, we can represent the functional logistic regression model as follows.

$$\log\frac{\pi_{i}}{1-\pi_{i}}\;=\;\beta_{0}\;+\;\overset{J}{\underset{\;j=1}{\sum }}\;z_{ij}^{T}b_{j}$$

The unknown parameters $b_{1},\ldots ,b_{J}$ in the model were estimated by the penalized likelihood method with an L2-type penalty, and cross-validation was performed using f1-score as the evaluation metric. The importance of each variable for prediction was assessed using the L2 norms of regression coefficients ${\hat{b}}_{j}=({\hat{b}}_{\;j1},\ldots ,{\hat{b}}_{jK}{)}^{T}$, and the coefficient functions were represented using these regression coefficients and corresponding eigenfunctions as ${\hat{\beta }}_{j}(t)={\hat{b}}_{j}^{T}u_{j}(t)$.

## 3 Results

**Figure 3:**
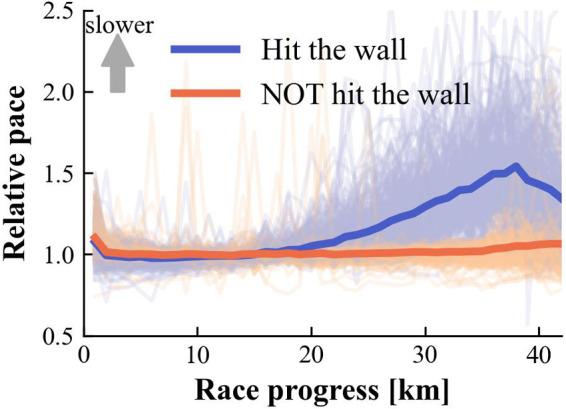
Comparison of relative pace profiles during marathon races between runners who experienced “hitting the wall” and those who did not. The thick blue and orange curves represent mean relative paces of runners who experienced and did not experience “hitting the wall”, respectively. Light-colored curves show individual runner data. Relative pace is expressed as a ratio to each runner's base pace (1.0 = average pace from 5 to 20 km); higher values indicate slower pace.

The relative pace profiles of runners who experienced “hitting the wall” during the race and those who did not are shown in Figure 3. The total number of runners who hit the wall was 306 (46.0%), while 359 (54.0%) runners did not. The slowing down of pace began at approximately 20 km, and the disparity between the two groups continued to widen until around 38 km.

When evaluated on the test dataset, the prediction model achieved a performance of 73.9% in accuracy, 75.8% in recall, 70.1% in precision, and 72.9% in F1-score. In other words, the model correctly identified about 7 out of every 10 runners, successfully retrieved 3 out of every 4 runners who actually classified as “hit the wall”, and delivered precise predictions for about 7 out of every 10 runners who predicted as “hit the wall”.

**Figure 4:**
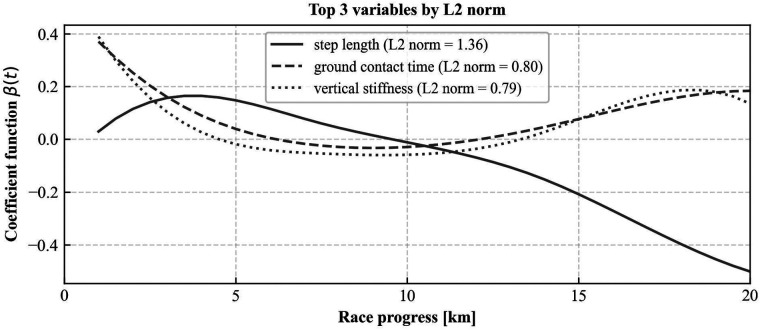
Coefficient functions of the 3 variables with the largest L2 norms in the functional logistic regression model.

Figure 4 shows the coefficient functions of the functional logistic regression model. In these coefficient functions, positive values indicate that runners are more likely to hit the wall when the corresponding running form variable is above its mean value at that point in the race, while negative values indicate a higher probability of hitting the wall when the variable is below its mean. The three variables presented in the upper graph correspond to those with the largest L2 norms, while those in the lower graph represent those with the smallest L2 norms. For step length, the coefficient function started with positive values at the beginning of the race but turned negative after approximately 10 km and continued a decreasing trend thereafter. Ground contact time and vertical stiffness exhibited positive values at the start, approached zero during the 5–10 km segment, and then showed an increasing trend again. These trends were associated with a higher likelihood of hitting the wall.

## 4 Discussion

The primary aim of this study was to predict significant pace reduction in the latter half of a full marathon based on biomechanical parameters observed during the first half of the race. Based on the information of running form in the first half of a full marathon, our functional logistic regression model successfully predicted the occurrence of significant slowing down in pace in the latter half of the race with the accuracy of more than 70%.

Our classification model revealed that severe pace reduction in the latter half of a full marathon can be predicted based on biomechanical parameters in the first half of the race. A previous study (23) constructed binary classification models to distinguish between pre- and post-fatigue states using IMU data during 400 m running at each participant's 5 km race pace. Their subject-independent model achieved 75% accuracy. While our model demonstrates similar classification performance, our approach offers a significant advancement in ecological validity by addressing several key differences in study design and population. Buckley et al. utilized a controlled fatiguing protocol (Beep Test) to induce fatigue. As Buckley et al. themselves acknowledged, this induced fatigue may be considered an unnatural way of fatiguing compared to the progressive decline in running economy over extended time and distance in an actual race. In contrast, our research examined biomechanical patterns during actual marathon races where “hitting the wall” naturally occurs in the real-world settings. Furthermore, our data collection in a natural marathon environment allowed for greater variety in race conditions and participant demographics than their sample of 21 recreational runners in a controlled track setting. These factors enhance the potential for practical application of our model across broader populations of runners and diverse race conditions.

Examination of coefficient functions revealed biomechanical parameters that significantly contribute to predicting pace reduction during marathons. Among these, step length, ground contact time, and vertical stiffness emerged as the most influential predictors. While running speed is fundamentally the product of step length and cadence, our analysis showed that step length patterns were more predictive of pace reduction than cadence. A recent meta-analysis (33) found that height-normalized step length showed stronger correlations with running economy (*r* = 0.27) compared to absolute stride length (*r* = 0.12) or cadence (*r* = −0.20). This suggests that height-normalized step length may better reflect individual running mechanics and fatigue states than absolute measurements. As fatigue progresses, runners experience reduced leg stiffness (19), compromising efficient elastic energy storage and increasing metabolic cost. This fatigue-induced reduction in leg stiffness likely manifests as shortened height-normalized step length, indicating impaired propulsive mechanics. Our findings suggest that these changes in height-normalized step length may more significantly impact running economy and pace than cadence alterations. While previous reviews have associated greater leg stiffness with improved running economy (19, 33), interpreting vertical stiffness derived from IMU data requires careful consideration. Higher calculated vertical stiffness might result from reduced knee flexion during ground contact. Tartaruga et al. (34) reported that less knee flexion at initial contact correlates with poorer running economy (*r* = −0.41). Therefore, elevated vertical stiffness in fatigued states might indicate inefficient force management rather than optimal elastic energy utilization, consequently affecting ground contact time.

The generalizability of our prediction model may be influenced by several confounding factors. Environmental conditions, such as ambient temperature and terrain gradient, are known to significantly impact running performance and biomechanics (35–38). Our current model does not explicitly account for these external variables, which could modulate how biomechanical parameters change under fatigue in different race environments. Furthermore, runner experience levels could be a critical factor. Highly trained athletes might exhibit different fatigue-induced biomechanical alterations and maintain their self-optimized stride patterns more effectively than recreational runners (19). Similarly, variations in race pacing strategies (e.g., even pacing vs. positive splits) can influence the onset of fatigue, potentially affecting the predictive power of biomechanical parameters captured in the first half of the race. Future models incorporating these contextual factors could enhance predictive accuracy and generalizability, though this would introduce increased data complexity.

The present study's approach—utilizing primarily biomechanical parameters and employing FPCA for efficient time-series data reduction, combined with interpretable logistic regression modeling—offers practical advantages for real-world applications in coaching contexts. For instance, runners can use this approach to analyze their half-marathon data so as to predict if they can maintain their pace during a full marathon, providing valuable insights for pre-race strategy development. Additionally, the biomechanical parameters identified through the model can help runners identify specific form improvements to mitigate their risk of “hitting the wall”. From a practical standpoint, a prediction improvement of even 5%–10% in classifying severe pace reduction could be meaningful for runners and coaches. Such an improvement could lead to more informed pacing decisions, targeted biomechanical interventions in daily training, and ultimately, a higher success rate in achieving marathon goals while potentially reducing injury risk. Even not perfect predictive capability can provide an objective assessment, allowing for adjustments in training load or race strategy that were previously based on subjective feeling alone. This shift from subjective perception to objective and data-driven insights represents a crucial step towards optimized marathon performance.

This study has several limitations that should be acknowledged. Firstly, our participants primarily consisted of Japanese runners. This may limit the generalizability of our findings to runners of different customs, body shapes, or training cultures. Secondly, the study might be subject to selection bias, as participants spontaneously purchased the IMU device, potentially leading to a sample that does not fully represent the broader marathon runner population. For instance, highly motivated or experienced runners might be represented, which could explain the lower proportion of runners who classified as “hit the wall” in our study compared to some previous research (5, 6). Moreover, while our prediction model successfully identified predictors of severe pace reduction, it did not categorize different magnitudes of slowing down in pace. Future research should address these limitations to enhance the external validity and applicability of the model across diverse running populations and race contexts. Besides, our current modelling approach does not allow for the identification of specific time windows within the first half of the race where predictive signals for pace reduction become more apparent.

Building upon the current findings, several opportunities for future research are recommended to enhance the model's predictive accuracy and practical utility. First, validation of the model in diverse populations from various regions and across different runner experience levels is essential to confirm its generalizability. Second, integrating additional physiological markers, such as heart rate data, and considering individual metabolic profiles or demographic factors like gender and personal best times may significantly improve predictive accuracy (6) and provide a more comprehensive understanding of the “hitting the wall” phenomenon. Furthermore, incorporating environmental conditions (e.g., ambient temperature, humidity) and course-specific factors (e.g., elevation changes) into the prediction model could account for external influences on biomechanical changes and pace reduction (35–38). Third, while this study utilized L2 norms to quantify the importance of coefficient functions for practical comparison, future methodological advancements should explore more nuanced quantitative measures of variable importance that could offer deeper practical interpretations for runners and coaches. Finally, future studies should explore the real-time implementation of such prediction models using wearable technology to provide immediate feedback to runners during training or races, potentially through smartphone applications. This would facilitate proactive adjustments to running strategy or form, enabling runners to mitigate the risk of severe pace reduction and achieve more consistent performance in endurance events.

## Data Availability

The data analyzed in this study is subject to the following licenses/restrictions: the datasets presented in this article are not readily available because of the privacy restrictions. Requests to access these datasets should be directed to Yosuke Miyazaki, yosuke.miyazaki@asics.com.
